# 

**Published:** 1878-10

**Authors:** 


### Table of Contents.

### ORIGINAL COMMUNICATIONS.

Introductory Lecture to the Course in Atlanta Medical College, for the Sessions of 1878-79. *By Prof. J. T. Banks*........ 385
The Causes of Premature Baldness. *By George H. Rohe, M.D., Atlanta*........ 391
Diphtheria; An Essay read before the Knox County (Tenn.) Medical Society. *By A. B. Tadlock, A.M., M.D.*........ 397

### REPORTS OF SOCIETIES.

Atlanta Academy of Medicine........ 407
Atlanta Medical College Medical Clinic........ 415

### SELECTIONS.

The Causes of Elephantiasis—a New Tinea........ 418

Irritable Testis........ 420

Consultation with Homœpaths and the Use of their Medicines........ 423

### EDITORIAL.

Quarantine........ 425
Cerebro-Spinal Meningitis........ 427
Atlanta Medical College........ 427

### CORRESPONDENCE.

European Correspondence........ 429

### BIBLIOGRAPHICAL.

Principles and Practice of Surgery........ 435
Anatomy, Descriptive and Surgical........ 436
The Antagonism of Therapeutic Agents........ 436

### EXCERPTA.

A Case of Transfusion........ 436
A New Treatment of Tapeworm........ 437
Therapeutic Value of Nitrate of Lead........ 437
Skin Grafting in the Colored Races........ 438
Skin Grafting........ 438
Treatment of Cancer by Bromine........ 439
Bromide of Potassium Externally as a Caustic and Hæmostatic........ 439
Catalepsy Produced by a Hair........ 439
Treatment of Diabetes by Salicin........ 440
Hay Fever........ 440
On Abscesses at the Verge of the Anus........ 440
Amputation of the Cervix Uteri........ 441
Threads of Whale Tendon as a Substitute for Catgut........ 441
Salicylic Acid as an Anaphrodisiac........ 441
Ipecac as a Hæmostatic........ 442
The Usefulness of Cremation........ 442
Consolidation of Medical Colleges........ 443
Spencer Wells' Ovariotomies........ 443
Causes of Suicide........ 443
The Dietic Value of Light Wines........ 444
Case of Poisoning by Oil of Bitter Almonds........ 444
Elastic Adhesive Plaster........ 446
Milk as a Vehicle for Quinine........ 447

